# Repeatability of self-report measures of physical activity, sedentary and travel behaviour in Hong Kong adolescents for the iHealt(H) and IPEN – Adolescent studies

**DOI:** 10.1186/1471-2431-14-142

**Published:** 2014-06-06

**Authors:** Ester Cerin, Cindy HP Sit, Ya-Jun Huang, Anthony Barnett, Duncan J Macfarlane, Stephen SH Wong

**Affiliations:** 1 Centre for Physical Activity and Nutrition Research, Deakin University, 221 Burwood Highway, Burwood VIC 3125, Australia; 2 Institute of Human Performance, The University of Hong Kong, Pokfulam Road, Hong Kong, Hong Kong SAR; 3 Department of Sports Science and Physical Education, The Chinese University of Hong Kong, Shatin, Hong Kong SAR

**Keywords:** Adolescents, Physical activity, Sedentary behaviour, Self-reports, Repeatability, Multi-country study, China

## Abstract

**Background:**

Physical activity and sedentary behaviour are important contributors to adolescents’ health. These behaviours may be affected by the school and neighbourhood built environments. However, current evidence on such effects is mainly limited to Western countries. The International Physical Activity and the Environment Network (IPEN)–Adolescent study aims to examine associations of the built environment with adolescent physical activity and sedentary behaviour across five continents.

We report on the repeatability of measures of in-school and out-of school physical activity, plus measures of out-of-school sedentary and travel behaviours adopted by the IPEN – Adolescent study and adapted for Chinese-speaking Hong Kong adolescents participating in the **i**nternational **H**ealthy **e**nvironments and **a**ctive **l**iving in **t**eenagers–(**H**ong Kong) [iHealt(H)] study, which is part of IPEN-Adolescent.

**Methods:**

Items gauging in-school physical activity and out-of-school physical activity, and out-of-school sedentary and travel behaviours developed for the IPEN – Adolescent study were translated from English into Chinese, adapted, and pilot tested. Sixty-eight Chinese-speaking 12–17 year old secondary school students (36 boys; 32 girls) residing in areas of Hong Kong differing in transport-related walkability were recruited. They self-completed the survey items twice, 8–16 days apart. Test-retest reliability was assessed for the whole sample and by gender using one-way random effects intra-class correlation coefficients (ICC). Test-retest reliability of items with restricted variability was assessed using percentage agreement.

**Results:**

Overall test-retest reliability of items and scales was moderate to excellent (ICC = 0.47–0.92). Items with restricted variability in responses had a high percentage agreement (92%-100%). Test-retest reliability was similar in girls and boys, with the exception of daily hours of homework (reliability higher in girls) and number of school-based sports teams or after-school physical activity classes (reliability higher in boys).

**Conclusions:**

The translated and adapted self-report measures of physical activity, sedentary and travel behaviours used in the iHealt(H) study are sufficiently reliable. Levels of reliability are comparable or slightly higher than those observed for the original measures.

## Background

Physical activity is a well-established modifiable factor that promotes health and prevents obesity in young people [1-3]. Sedentary behaviour, defined as sitting or lying activities associated with low levels of energy expenditure, has recently been identified as an important contributor to poor cardio-metabolic health [4] and adiposity [5,6].

Studies indicate that the built environment is one of the main sources of factors shaping physical activity and sedentary behaviour in youth [7,8]. Built environments particularly important to youth activity habits include schools and the residential neighbourhood [9-14]. Yet, most studies examining environmental correlates of youth activity habits have been conducted in Western countries with limited environmental variability. To more accurately capture the dose–response relationships of the environment with physical activity and sedentary behaviours, it is important to maximise the variability of exposure (built environment) measures. This is because restricted variability results in the underestimation of the strength of linear relationships or the inability to detect curvilinear relationships [15,16].

By collecting data from locations across five continents, the International Physical Activity and the Environment Network (IPEN)–Adolescent study aims to accurately estimate associations of the environment with physical activity and sedentary behaviour in adolescents (http://www.ipenproject.org/IPEN_adolescent.html). The iHealt(H) [international Healthy environments and active living in teenagers–(Hong Kong)] study is part of IPEN-Adolescent. As the measures for IPEN-Adolescent originated in the USA, it is important to establish the metric properties of the corresponding measures translated and adapted for other study sites. We report on the repeatability of measures of in-school and out-of-school physical activity, and out-of-school sedentary and travel behaviours translated and adapted for Chinese-speaking Hong Kong adolescents participating in the iHealt(H) study. As Hong Kong represents the IPEN–Adolescent study site that differs the most from the location where the original measures were developed (USA), it is particularly important to assess the metric properties of these measures here.

## Methods

### Participants and procedures

This study was approved by the Human Research Ethics Committee for Non-Clinical Faculties of The University of Hong Kong. Two schools, one located in a high walkable and the other in a low walkable urban area of Hong Kong, were contacted and consented to participate in the study. “Walkability” was defined using Geographic Information Systems measures of dwelling density and street connectivity [17]. We recruited participants from high and low walkable areas to maximize the variability in transport-related behaviour (active vs. motorized).

Designated school staff screened the students for eligibility and invited them to participate in the study. We aimed to recruit 68 participants balanced by gender and age groups (i.e., 12–13, 14–15 and 16–17 year-olds). Power calculations indicated that this would allow detection of an intra-class correlation coefficient (ICC, a measure of test-retest reliability) of 0.40 (corresponding to minimally acceptable value) in each gender group (n = 34) with 80% power while adopting a probability level of 0.05 [18]. Eligibility criteria were being a 12–17 year old secondary school student (the target age range of the IPEN–Adolescent and iHealt(H) studies), being able to read and write in Chinese, residing in a particular pre-selected area, having lived at the current address for at least six months, and not suffering from a disability/illness that precluded engagement in moderate-intensity physical activity. Parental/caregiver’s consent and student’s assent were obtained prior to participation in the study.

The final sample consisted of 68 participants (36 boys; 32 girls; mean age: 15.4 years; response rate: 57%). In November 2011, students self-completed the surveys in their free time indicating the date of completion at the end of the survey. They were given a phone number that they could call in case they needed clarification or assistance with the completion of the survey. Students returned the completed survey to the schools and were given the second survey 7–10 days after the date of completion noted in the first survey. The average time of completion between the first and second surveys was 13 days (range 8 to 16 days). This test-retest time interval was chosen for two reasons. Firstly, it was used in the test-retest reliability assessment of the original measures [19]. Secondly, it is generally maintained that the average test-retest time interval for physical activity and sedentary behaviour measures recalling behaviours performed in the previous week should not exceed 14 days [20]. Participants were offered a HK$ 50 voucher (participation incentive) upon successful completion of both surveys.

### Measures

#### Physical activity in a school setting

Opportunities to engage in school-based physical activity were measured by assessing the time spent on physical education (PE) classes and recess in a usual week. This was done by asking students to indicate the weekly frequency and average duration (in minutes) of recess periods and PE classes. Frequency and duration measures were multiplied to obtain an estimate of total weekly minutes of recess and PE class time. Students also reported the number of sports teams or after-school physical activity classes they participated in *at school* in the past year, with responses ranging from ‘0’ to ‘4 or more’. These items were derived from the Active Where? [21] and Teen Environment And Neighborhood (TEAN) studies [22], with the original versions of these measures showing substantial-to-excellent test-retest reliability (ICC = 0.70-0.86) [23] (see Additional file 1).

#### Out-of-school physical activity and sedentary behaviours

Out-of-school, leisure-time moderate-to-vigorous physical activity (MVPA) was assessed using a two item scale developed by Prochaska and colleagues [19]. MVPA was defined as producing increases in heart rate and breathing some of the time, with examples given to explain the concept (i.e., engagement in running, brisk walking, biking, dancing, etc.). The two items asked students to report the number of days on which they were physically active for a total of at least 60 minutes per day “over the past 7 days” and “on a typical or usual week”. Typically, the responses on these two items are averaged. Test-retest reliability of the original English versions of the two items and their composite measure was moderate (0.57-0.63) [23,24]. We also assessed out-of-school participation in sports teams and physical activity classes using the item ‘In the past year, how many sports teams or physical activity classes have you participated in outside of school?’. This item was adopted from the TEAN study [22], with possible responses ranging from ‘0’ to ‘4 or more’. The test-retest reliability of this item had not been previously assessed.

Out-of-school sedentary behaviour *on a typical school day* (i.e., not on weekends) was measured using six items of a scale that showed good reliability and validity with adolescents (ICC = 0.67 in the Active Where study) [24]. Items include duration of watching TV/videos/DVDs; playing sedentary computer or video games; using internet, emailing or other electronic media for leisure; doing homework; reading a book or magazine not for school; and riding in a car, bus or other vehicle. Responses were on a 7-point scale ranging from ‘0 hours’ to ‘4 or more hours per day’. A score for total sedentary time was computed by summing the single-item responses converted into hours (e.g., the response ’15 min per day’ was assigned a value of 0.25 hours). We also computed a score for total screen time by summing the weighted responses (with weights corresponding to number of hours: e.g., ’30 min per day’ was assigned a value of 0.50 hours) on the three relevant items (watching TV/video/DVD; Playing sedentary computer or video games; Using internet/emailing/other electronic media for leisure).

#### Transportation behaviour

Weekly frequency of walking or cycling to/from nine types of destinations (in the past year) was assessed using items from Active Where? [23]. Response options ranged from ‘never’ to ‘4 or more times per week’. A total score of active transportation (excluding walking/cycling trips to/from school) was created by summing the single-item responses converted into frequency per week (e.g., the response ‘once a month or less’ was assigned a value of 0.25 times per week). The test-retest reliability of the original items had not been evaluated.

The frequency of using six modes of transport (walking; cycling; public transport; taxi; school bus; family car) to travel to/from school in an average school week was gauged using a scale adapted from Active Where? [23]. To reflect the whole range of transportation options in Hong Kong, the original three-mode scale (walking; cycling; car or bus) was expanded to include taxies, and school buses. Additionally, it was necessary to change the item “bus” to “public transport” to include public transport modes other than buses that are available in Hong Kong (e.g., tram, train, ferry and the Mass Transit Railway). These modifications have now been included in the IPEN–Adolescent study protocol to provide a better coverage of alternative modes of transport that adolescents may use to commute to/from school in various geographical locations. Response options ranged from 0 to 5 days. A composite score of active transportation to/from school was created by summing the responses on the ‘walking’ and ‘cycling’ items (max score 10 trips per week). Test-retest reliability (ICC) of the original single items ranged from 0.51 to 0.84, and that of the total active commuting to/from school score was 0.83 [23].

All original measures were translated in Chinese and back-translated in English following the World Health Organization guidelines [25]. Any discrepancies between the original and back-translated versions of the questionnaires were iteratively reviewed, discussed and resolved by a panel of experts including bilingual (Chinese- and English-speaking) members with expertise in measurement of physical activity and sedentary behaviours and extensive experience in questionnaire development and cross-cultural adaptation. The final working version of the survey was pilot tested on a small group of Chinese-speaking university and secondary school students for clarity. Secondary school students took, on average, 9 minutes (SD = 3) to complete the measures included in this study. Pilot testing resulted in minor amendments to questionnaire instructions, the final versions of which were found to be clear.

#### Data analyses

Descriptive statistics (means and standard deviations) were computed for each item and scale (composite scores) for the whole sample and by respondents’ gender. Test-retest reliability of each item and scale was established by computing one-way random effects ICCs for the whole sample and by respondents’ gender. Non-normally distributed, positively skewed variables were log-transformed before computing ICCs. Based on previously proposed classification systems, ICC values below 0.40 were classified as poor, 0.41 to 0.60 as moderate, 0.61 to 0.80 as substantial and over 0.80 as excellent reliability [26]. Percent agreement was computed for items with ordinal responses. This was particularly necessary for items with restricted variability in responses for which it was not possible to compute an ICC. Items and scales with restricted variability and percent agreement greater than 80% were considered to have good reliability [27]. The significance of the between-gender difference in test-retest reliability estimates was tested using bootstrap methods, whereby 95% bootstrapped confidence intervals of differences between ICCs excluding 0 were considered statistically significant at the two-tailed probability level of 0.05. All analyses were conducted in R.

## Results

Table 1 shows the means and standard deviations of scores on all items and composite scores of the scales used in this study. Students reported engaging in PE lessons for approximately 75 minutes per week, and around 100 minutes of recess time per week. The average number of days per week of engagement in more than 60 minutes of MVPA was low (< 2). On average, students reported engaging in over 5 hours of sedentary behaviours outside school per day, with the largest amount of time spent on homework activities. On average, respondents reported almost seven walking or cycling trips to/from destinations other than school per week, with trips to public transportation stops and food outlets being the most frequent. The most common modes of transport to and from school were public transport and walking, with students undertaking 4.0 active trips to/from school per week.

**Table 1:** Descriptive statistics of physical activity, sedentary and travel behaviour self-report measures for Hong Kong adolescents

**Item/Measure [theoretical range]**	**Overall sample (N = 68)**	**Boys (n = 36)**	**Girls (n = 32)**
	**Mean (SD)**	**Mean (SD)**	**Mean (SD)**
**Setting/domain: SCHOOL**			
**Physical Education (PE)**			
PE classes (days/week) [0–5]	1.3 (0.7)	1.4 (0.8)	1.3 (0.6)
Length of PE period (min/class)	58.2 (18.8)	57.7 (17.6)	58.7 (20.0)
***Total PE time (min/week)***	74.3 (45.3)	77.3 (53.2)	71.5 (37.3)
Recess at school (days/week) [0–5]	4.8 (0.8)	4.7 (1.0)	4.9 (0.5)
Total length of recess (min/day)	21.3 (9.6)	20.8 (8.6)	21.7 (10.5)
***Total recess time (min/week)***	104.0 (50.6)	100.7 (46.0)	106.9 (53.9)
Number of sports teams or after-school physical activity classes at school in past year [0–4]	1.1 (0.9)	1.2 (0.9)	0.9 (0.8)
**Setting/domain: OUT-OF-SCHOOL**			
**Leisure-time physical activity (PA)**			
≥ 60 min/day of out-of-school PA in past 7 days (days/week) [0–7]	1.8 (2.0)	2.0 (2.2)	1.1 (1.8)
≥ 60 min/day of out-of-school PA in usual week (days/week) [0–7]	2.0 (2.0)	2.4 (2.2)	1.6 (1.8)
***Average score on two out-of-school PA [0–7]***	1.9 (1.8)	2.2 (2.0)	1.5 (1.7)
Number of sports teams or after-school physical activity classes out-of-school in past year [0–4]	0.9 (1.0)	0.9 (1.0)	0.9 (1.0)
**Sedentary behaviour (hours/day) [0–4]**			
Watching TV/videos/DVD	1.1 (1.0)	1.0 (0.8)	1.1 (1.1)
Playing sedentary computer or video games	0.6 (0.8)	0.8 (0.9)	0.3 (0.6)
Using internet/emailing/other electronic media for leisure	0.7 (0.7)	0.5 (0.5)	0.8 (0.8)
Doing homework	1.8 (1.1)	1.7 (1.1)	1.9 (1.2)
Reading a book or magazine (not for school)	0.6 (0.9)	0.6 (0.9)	0.7 (0.8)
Riding in a car, bus, etc.	0.6 (0.9)	0.7 (0.7)	0.6 (0.6)
***Total screen time (hours/day) [0–12]***	2.3 (1.7)	2.3 (1.4)	2.3 (1.9)
***Total sedentary time (hours/day) [0–24]***	5.3 (2.5)	5.2 (2.3)	5.4 (2.6)
**Setting/domain: TRANSPORTATION**			
**Walking or cycling to/from destinations (times/week) [0–4]**			
Indoor or exercise facility	0.5 (0.8)	0.5 (0.8)	0.5 (0.8)
Friend’s or relative house	0.5 (0.8)	0.4 (0.8)	0.5 (0.8)
Outdoor recreation place	0.6 (0.9)	0.7 (0.9)	0.6 (0.9)
Food store or restaurant/café	1.5 (1.5)	1.6 (1.5)	1.4 (1.4)
Other retail stores	1.1 (1.3)	1.0 (1.2)	1.2 (1.3)
Non-school social or educational activities	0.5 (0.8)	0.5 (0.9)	0.5 (0.7)
Public transportation stop	2.3 (1.7)	2.2 (1.7)	2.4 (1.6)
Work	0.0 (0.0)	0.0 (0.0)	0.0 (0.0)
Others	0.0 (0.0)	0.0 (0.0)	0.0 (0.0)
***Total score on walking or cycling to/from destinations (times/week) [0–28]***	6.9 (4.5)	6.9 (4.7)	7.0 (4.2)
**Transportation modes to/from school (days/week) [0–5]**			
To school			
Walk	1.9 (2.3)	1.4 (2.2)	2.3 (2.4)
Bicycle	0.1 (0.3)	0.1 (0.4)	0.0 (0.0)
Public transport	3.0 (2.3)	3.2 (2.2)	2.8 (0.2)
Taxi	0.1 (0.3)	0.1 (0.4)	0.0 (0.0)
School bus	0.1 (0.3)	0.1 (0.4)	0.0 (0.0)
Family car	0.4 (1.3)	0.7 (1.6)	0.2 (0.7)
From school			
Walk	2.1 (2.3)	1.7 (1.6)	2.6 (2.3)
Bicycle	0.1 (0.6)	0.2 (0.8)	0.0 (0.0)
Public transport	3.1 (2.3)	3.5 (2.1)	2.7 (2.3)
Taxi	0.1 (0.3)	0.1 (0.4)	0.0 (0.0)
School bus	0.1 (0.3)	0.1 (0.4)	0.0 (0.0)
Family car	0.1 (0.2)	0.1 (0.2)	0.0 (0.0)
***Total frequency of active transportation to/from school (times/week) (0–10)***	4.0 (4.5)	3.2 (4.2)	4.8 (4.6)

Overall, test-retest reliability of the items and scales included in this study was moderate to excellent (Table 2). Several items showed restricted variability in responses (number of PE classes per week; days per week of recess at school; bicycle, taxi, school bus trips to/from school). Nevertheless, for these items, the percentage agreement in responses between the first and second assessments was very high (92%-100%). Items gauging modes of transportation to/from school showed the highest level of reliability. These were followed by scales/items gauging physical activity and sedentary behaviours. The items/scale assessing active transportation to/from destinations other than schools tended to display the lowest levels of reliability, with the exception of those gauging frequency of trips to public transportation stops and food outlets.

**Table 2:** Test-retest reliability of physical activity, sedentary and travel behaviour self-report measures for Hong Kong adolescents

**Item/Measure**	**Overall sample (N = 68)**	**Boys (n = 36)**	**Girls (n = 32)**
**Setting/domain: SCHOOL**			
**Physical Education**			
PE classes (days/week)	NC (98%)	NC (96%)	NC (100%)
Length of PE period (min/class)	0.89	0.94	0.86
***Total PE time (min/week)***	0.84	0.87	0.81
**Recess**			
Recess at school (days/week)	NC (94%)	NC (92%)	NC (98%)
Total length of recess (min/day)	0.69	0.64	0.72
***Total recess time (min/week)***	0.72	0.67	0.76
Number of sports teams or after-school physical activity classes at school in past year	0.74 (79%)	0.84 (89%)	0.65* (69%)
**Setting/domain: OUT-OF-SCHOOL**			
**Leisure-time physical activity (PA)**			
≥ 60 min/day of out-of-school PA in past 7 days (day/week)	0.70 (76%)	0.69 (69%)	0.72 (84%)
≥ 60 min/day of out-of-school PA in usual week (day/week)	0.79 (65%)	0.80 (67%)	0.78 (63%)
***Average score on two out-of-school PA***	0.76	0.74	0.77
Number of sports teams or after-school physical activity classes out-of-school in past year	0.89 (90%)	0.90 (92%)	0.87 (88%)
**Sedentary behaviour (hours/day)**			
Watching TV/videos/DVD	0.62 (74%)	0.61 (64%)	0.64 (78%)
Playing sedentary computer or video games	0.66 (72%)	0.65 (69%)	0.68 (75%)
Using internet/emailing/other electronic media for leisure	0.58 (65%)	0.59 (67%)	0.56 (63%)
Doing homework	0.78 (76%)	0.69 (67%)	0.85* (88%)
Reading a book or magazine (not for school)	0.61 (62%)	0.54 (57%)	0.67 (69%)
Riding in a car, bus, etc.	0.51 (68%)	0.52 (69%)	0.49 (67%)
***Total screen time (hours/day)***	0.62	0.61	0.63
***Total sedentary time (hours/day)***	0.65	0.62	0.67
**Setting/domain: TRANSPORTATION**			
**Walking or cycling to/from destinations (times/week)**			
Indoor or exercise facility	0.61 (76%)	0.55 (72%)	0.67 (81%)
Friend’s or relative house	0.48 (57%)	0.49 (58%)	0.45 (56%)
Outdoor recreation place	0.47 (62%)	0.47 (61%)	0.46 (63%)
Food store or restaurant/cafe	0.82 (80%)	0.78 (75%)	0.86 (84%)
Other retail stores	0.51 (62%)	0.46 (58%)	0.57 (66%)
Non-school social or educational activities	0.51 (68%)	0.52 (67%)	0.51 (69%)
Public transportation stop	0.71 (69%)	0.71 (69%)	0.71 (69%)
Work	NA (100%)	NA (100%)	NA (100%)
Other	NA (100%)	NA (100%)	NA (100%)
***Total score on walking or cycling to/from destinations (times/week)***	0.59	0.56	0.62
**Transportation modes to/from school (days/week) [0–5]**			
To school			
Walk	0.89 (90%)	0.87 (89%)	0.91 (91%)
Bicycle	NC (100%)	NC (100%)	NC (100%)
Public transport	0.92 (97%)	0.92 (97%)	0.91 (97%)
Taxi	NC (100%)	NC (100%)	NC (100%)
School bus	NC (100%)	NC (100%)	NC (100%)
Family car	0.92 (96%)	0.89 (94%)	0.94 (97%)
From school			
Walk	0.76 (79%)	0.73 (72%)	0.79 (88%)
Bicycle	NC (100%)	NC (100%)	NC (100%)
Public transport	0.68 (74%)	0.71 (78%)	0.66 (69%)
Taxi	NC (100%)	NC (100%)	NC (100%)
School bus	NC (100%)	NC (100%)	NC (100%)
Family car	NC (99%)	NC (97%)	NC (100%)
***Total frequency of active transportation to/from school (times/week)***	0.88	0.86	0.90

*Notes.* *Significant difference between genders (p < .05); NC = intra-class correlation coefficient could not be computed due to lack of variability in responses. Percent agreement is in brackets. All other values represent intra-class correlation coefficients. Percent agreement not computed for continuous measures.

In general, test-retest reliability was similar in girls and boys. Girls (ICC = 0.65) obtained a lower test-retest reliability coefficient than boys (ICC = 0.84) for number of sports teams or after-school physical activity classes at school in the past year (Table 2). The opposite was true for the item assessing daily hours spent on homework (reliability higher in girls, 0.85, than boys, 0.69).

## Discussion

The iHealth(H) study employs self-report outcome measures originally developed for US adolescents [19,21-23] that have been translated and adapted for Chinese-speaking Hong Kong adolescents. The main aim of this study was to assess the repeatability of the physical activity, sedentary and travel behaviour measures included in the iHealt(H) study and compare them to those observed in US samples.

The levels of test-retest reliability of measures of physical activities in a school setting were substantial to excellent, mirroring findings on an US sample of adolescents [22]. In general, measures of recess time tended to have lower reliability than those of PE time possibly due to the PE lessons being less flexible and less variable from week to week than the recess periods. It is also interesting that the reliability of a measure of participation in school-based sports teams and physical activities after school was significantly lower, although still substantial, in girls than in boys. Higher levels (see Table 1) and more regular participation and interest in such activities among boys may explain these gender differences [28].

Excellent reliability was observed for participation in out-of-school sports teams and physical activities. Also the reliability of measures of leisure-time physical activities was substantial and higher than that found in US samples [19,23]. Perhaps this is due to Hong Kong adolescents having more structured time schedules and physical activities than their US counterparts, and adolescents in the US having more freedom of action and a greater variety of physical activities to choose from. Hong Kong parents and society are notorious for the importance they give to academic achievement [29]. This may limit the opportunities and time that Hong Kong adolescents have for physical activities [30], which then need to become more planned and structured to fit into their busy academic/tutorial schedules after school.

In general, and in line with findings observed in the US, the reliability coefficients of out-of-school sedentary behaviour measures were lower than those of measures of structured physical activities. Apart from homework, which was the out-of-school sedentary activity with the highest level of reliability, the activities included in the measure of sedentary behaviour were leisure-time, discretionary activities and, as such, were unlikely to be as regular and temporally-defined as structured activities. Interestingly, girls showed substantially higher levels of reliability for time spent on homework than did boys. There is some evidence that adolescent girls tend to be more disciplined and academically-oriented than boys [31] and thus may more rigorously adhere to after-school homework schedules.

The reliability of items measuring walking/cycling to destinations was moderate to substantial but lower than that of measures of structured physical activities and out-of-school sedentary time. This is not surprising since the former activities were, in general, less frequent and likely more sporadic than the latter (see Table 1). Other studies have found measures of structured and leisure-time activities to be more reliable than measures of active transport [32]. The assumption that frequency and regularity of a specific physical activity may be positively associated with the reliability of its measure is also supported by the fact that in this study, the two destinations with the highest reliability (public transport stop and food store or restaurant/café) were also the destinations most frequently visited (Tables 1 and 2). Unfortunately, the reliability of the original items measuring walking/cycling to destination has not been assessed. Hence, since this is the first study to report reliability estimates for this measure, our findings cannot be compared to those from the US as we have done for measures of sedentary behaviours and structured physical activity.

The reliability of items assessing transport modes used to commute to/from school was substantial to excellent. Also, the reliability of total frequency of active transportation to/from school was higher than in the US sample [22], possibly due to the lower prevalence such activity in US adolescents as compared to their Hong Kong counterparts [33]. It is interesting that items assessing travel to school tended to have higher reliability than those assessing travel from school. The mode of transport to school may be less variable due to students going to school at the same time of the day, whilst the mode of transport used to travel from school may be more variable and depend on the duration of school activities and nature of after-school activities on a particular day.

This study has several strengths and limitations. Strengths include: the systematic recruitment of students residing in high and low walkable areas (relative to Hong Kong standards); the analysis of test-retest reliability of measures by participants’ gender; and this study being the first to report on test-retest reliability of several measures of travel behaviour and out-of-school leisure-time physical activity that are being used in the IPEN–Adolescent study examining associations of the built environment and physical activity in adolescents in several countries across the globe. The fact that the relatively small sample size did not allow an analysis of differences in test-reliability by age groups (e.g., 12–14 vs. 15–17 year olds) is a study limitation. Although 12-13-year, 14-15-year and 16-17-year age groups were balanced, most participants falling into the 14-15-year age category were 15-year-olds. This resulted in getting too small a 12-14-year-old subgroup of participants (n = 27) for a sufficiently robust estimation of age-specific test-retest reliability. Another limitation pertains to the restricted variability in the responses of several items assessing mode of transport, which did not allow a more robust assessment of these items’ repeatability. Finally, this study did not assess the validity of the examined measures. Future studies will need to determine whether the total self-reported time spent on a specific type of behaviour assessed using multiple items (e.g., active transportation to various destinations) yields biased estimates (e.g., over-reporting) of that behaviour.

## Conclusions

In conclusion, this study indicates that the levels of reliability of the self-report measures of physical activity, sedentary and travel behaviours used in the iHealt(H) study range from moderate to excellent and are comparable if not greater than those of the original English version of the respective measures. Hence, these self-report instruments can reliably collect data on physical activity, sedentary and travel behaviours in Hong Kong adolescents. Overall these positive findings suggest that the measures used in this study could potentially be successfully translated and adapted for use with other populations of Asian adolescents. Future studies will need to address this issue as well as establish the validity of these measures.

## Abbreviations

ICC: Intra-class correlation coefficients; iHealt(H): international Healthy **e**nvironments and active **l**iving in teenagers – (Hong Kong); IPEN: **I**nternational Physical Activity and the Environment Network; PE: Physical education; MVPA: Moderate-to-vigorous physical activity; TEAN: Teen environment and neighborhood.

## Competing interests

The author(s) declare that they have no competing interests.

## Authors’ contributions

EC is the principal investigator of the iHealt(H) and Hong Kong component of the IPEN–Adolescent studies. She conceptualised the iHealt(H) study and this paper, conducted the analyses and drafted the manuscript. CHPS and YJH contributed to the translation and adaptation of the original measures and to the conceptualisation of the iHealt(H) study. AB, DJM and SHSW contributed to the conceptualisation of the iHealt(H) study. AB, CHPS, YJH and SHSW helped with the coordination of the data collection. All authors critically reviewed various drafts of the manuscript and approved its final version.

## Pre-publication history

The pre-publication history for this paper can be accessed here:

http://www.biomedcentral.com/1471-2431/14/142/prepub

## Supplementary Material

Additional file 1 Physical Activity and Sedentary Behaviour Measures used in the iHealt(H). Click here for file
