# Supplementary material for: Repeatability of self-report measures of physical activity, sedentary and travel behaviour in Hong Kong adolescents for the iHealt(H) and IPEN – Adolescent studies
Source: BMC Pediatr. 2014 Jun 6;14:142. doi: 10.1186/1471-2431-14-142 (PMC4060092; doi:10.1186/1471-2431-14-142)
Supplement: Additional file 1 — Physical Activity and Sedentary Behaviour Measures used in the iHealt(H). [file 1471-2431-14-142-S1.docx]

**Physical Activity and Sedentary Behaviour Measures used in the iHealt(H) study**

**Setting/domain: SCHOOL**

Physical Education

| How many days per week do you have gym or Physical Education (PE) class at school? | | | | | |
| --- | --- | --- | --- | --- | --- |
| 0 days | 1 day | 2 days | 3 days | 4 days | 5 days |
| If you have PE, on average, how long is each PE period? | | | | | |
| _______minutes per class | | |  | | |

Recess

| How many days per week do you have recess at school? | | | | | |
| --- | --- | --- | --- | --- | --- |
| 0 days | 1 day | 2 days | 3 days | 4 days | 5 days |
| If you have recess, on average, how long is the total time spent in recess? | | | | | |
| _______minutes per class | | |  | | |

Sports teams and after-school physical activity classes

| In the past year, how many sports teams or “after school” physical activity classes (not PE) have you participated in at school? If you play for more than 1 team of the same sport or across 2 seasons in the same year, count this as 2. | | | | |
| --- | --- | --- | --- | --- |
| 0 | 1 | 2 | 3 | 4 or more |

**Setting/domain: OUT-OF-SCHOOL**

Leisure-time physical activity

| Physical activity is any activity that increases your heart rate and makes you get out of breath at least some of the time. Physical activity can be done in sports, being active with friends or walking to school. Examples of physical activity are running, brisk walking, rollerblading, biking, dancing, skateboarding, swimming, soccer, basketball, football and badminton.  Over the past seven days, on how many days were you physically active for a total of at least 60 minutes per day (do not include school PE or gym class)? | | | | | | | |
| --- | --- | --- | --- | --- | --- | --- | --- |
| 0 days | 1 day | 2 days | 3 days | 4 days | 5 days | 6 days | 7 days |
| Over a typical or usual week, on how many days are you physically active for a total of at least 60 minutes per day (do not include school PE or gym class)? | | | | | | | |
| 0 days | 1 day | 2 days | 3 days | 4 days | 5 days | 6 days | 7 days |

Sports teams and after-school physical activity classes

In the past year, how many sports teams or physical activity classes have you participated in outside of school? If you play for more than 1 team of the same sport or across 2 seasons in the same year, count this as 2.

0 1 2 3 4 or more

Sedentary behaviour

Sedentary behaviours are activities that generally involve sitting and not moving around, like watching TV, playing video games, reading, etc.

| Please indicate how much time on a typical school day you do the following activities. Please think about the time from when you wake up until you go to bed. Please DO NOT include time when you are in school during regular hours. Do not include weekends. | | | | | | | |
| --- | --- | --- | --- | --- | --- | --- | --- |
|  | None | 15 min per day | 30 min per day | 1 hour per day | 2 hours per day | 3 hours per day | 4 or more hours per day |
| 1. Watching television/videos/DVD’s | 0 | 1 | 2 | 3 | 4 | 5 | 6 |
| 2. Playing sedentary computer or video games (like Nintendo or Xbox) | 0 | 1 | 2 | 3 | 4 | 5 | 6 |
| 3. Using the internet, emailing or other electronic media for leisure | 0 | 1 | 2 | 3 | 4 | 5 | 6 |
| 4. Doing homework (including reading, writing or using the computer) | 0 | 1 | 2 | 3 | 4 | 5 | 6 |
| 5. Reading a book or magazine NOT for school (including comic books) | 0 | 1 | 2 | 3 | 4 | 5 | 6 |
| 6. Riding in a car, bus, etc. | 0 | 1 | 2 | 3 | 4 | 5 | 6 |

**Setting/domain: TRANSPORTATION**

Walking or cycling to/from destinations

Remember, think about the PAST YEAR.

| How often do you usually **walk or bike** to/from the following? | | | | | | |
| --- | --- | --- | --- | --- | --- | --- |
|  | Never | Once a month or less | Once every other week | Once a week | 2 or 3 times per week | 4 or more times per week |
| 1. Indoor recreation or exercise facility (public or private; dance, martial arts) | 0 | 1 | 2 | 3 | 4 | 5 |
| 2. Friend’s or relative’s house | 0 | 1 | 2 | 3 | 4 | 5 |
| 3. Outdoor recreation place (park, sports field, open space, creek) | 0 | 1 | 2 | 3 | 4 | 5 |
| 4. Food store or restaurant/cafe | 0 | 1 | 2 | 3 | 4 | 5 |
| 5. Other retail stores (e.g., music, clothes) | 0 | 1 | 2 | 3 | 4 | 5 |
| 6. Non-school social or educational activities (e.g., church group, band) | 0 | 1 | 2 | 3 | 4 | 5 |
| 7. Public transportation stop (bus, train, light rail) | 0 | 1 | 2 | 3 | 4 | 5 |
| 8. Work (check if not applicable □ ) | 0 | 1 | 2 | 3 | 4 | 5 |
| 9. Other: (please specify)  ____________________________ | 0 | 1 | 2 | 3 | 4 | 5 |

Transportation modes to/from school

| In an average school week, on how many days do you use the following modes of transportation to get to and from school? | | | | | | |
| --- | --- | --- | --- | --- | --- | --- |
| Days per week **TO** school: | 0 days | 1 day | 2 days | 3 days | 4 days | 5 days |
| 1. Walk | 0 | 1 | 2 | 3 | 4 | 5 |
| 2. Bicycle | 0 | 1 | 2 | 3 | 4 | 5 |
| 3. Public transport | 0 | 1 | 2 | 3 | 4 | 5 |
| 4. Taxi | 0 | 1 | 2 | 3 | 4 | 5 |
| 6. School bus | 0 | 1 | 2 | 3 | 4 | 5 |
| 7. Family car | 0 | 1 | 2 | 3 | 4 | 5 |
| Days per week **FROM** school: | 0 days | 1 day | 2 days | 3 days | 4 days | 5 days |
| 1. Walk | 0 | 1 | 2 | 3 | 4 | 5 |
| 2. Bicycle | 0 | 1 | 2 | 3 | 4 | 5 |
| 3. Public transport | 0 | 1 | 2 | 3 | 4 | 5 |
| 4.Taxi | 0 | 1 | 2 | 3 | 4 | 5 |
| 5. School bus | 0 | 1 | 2 | 3 | 4 | 5 |
| 6. Family car | 0 | 1 | 2 | 3 | 4 | 5 |
